# Radiomic Analysis for Pretreatment Prediction of Recurrence Post-Radiotherapy in Cervical Squamous Cell Carcinoma Cancer

**DOI:** 10.3390/diagnostics12102346

**Published:** 2022-09-28

**Authors:** Daisuke Kawahara, Ikuno Nishibuchi, Masashi Kawamura, Takahito Yoshida, Iemasa Koh, Katsuyuki Tomono, Masaki Sekine, Haruko Takahashi, Yutaka Kikuchi, Yoshiki Kudo, Yasushi Nagata

**Affiliations:** 1 Department of Radiation Oncology, Graduate School of Biomedical and Health Sciences, Hiroshima University, Hiroshima 734-8551, Japan; 2 Medical and Dental Sciences Course, Graduate School of Biomedical and Health Sciences, Hiroshima University, Hiroshima 734-8551, Japan; 3 School of Medicine, Hiroshima University, Hiroshima 734-8551, Japan; 4 Department of Obstetrics and Gynecology, Graduate School of Biomedical and Health Sciences, Hiroshima University, Hiroshima 734-8551, Japan; 5 Graduate School of Integrated Sciences for Life, Hiroshima University, Kagamiyama 1-3-1, Higashi-Hiroshima, Hiroshima 739-8526, Japan; 6 Hiroshima High-Precision Radiotherapy Cancer Center, Hiroshima 732-0057, Japan

**Keywords:** radiotherapy, machine learning, cervix cancer

## Abstract

Background: The current study aims to predict the recurrence of cervical cancer patients treated with radiotherapy from radiomics features on pretreatment T1- and T2-weighted MR images. Methods: A total of 89 patients were split into model training (63 patients) and model testing (26 patients). The predictors of recurrence were selected using the least absolute shrinkage and selection operator (LASSO) regression. The machine learning used neural network classifiers. Results: Using LASSO analysis of radiomics, we found 25 features from the T1-weighted and 4 features from T2-weighted MR images, respectively. The accuracy was highest with the combination of T1- and T2-weighted MR images. The model performances with T1- or T2-weighted MR images were 86.4% or 89.4% accuracy, 74.9% or 38.1% sensitivity, 81.8% or 72.2% specificity, and 0.89 or 0.69 of the area under the curve (AUC). The model performance with the combination of T1- and T2-weighted MR images was 93.1% accuracy, 81.6% sensitivity, 88.7% specificity, and 0.94 of AUC. Conclusions: The radiomics analysis with T1- and T2-weighted MR images could highly predict the recurrence of cervix cancer after radiotherapy. The variation of the distribution and the difference in the pixel number at the peripheral and the center were important predictors.

## 1. Introduction

Cervical cancer is one of the most common malignant tumors in women worldwide [1]. Definitive radiotherapy is the mainstream treatment for cervical squamous cell carcinoma in early-stage and advanced cases. The treatment outcomes of radiotherapy and surgery are comparable in the early stage. For locally advanced-unresectable cervix cancer, concurrent chemoradiotherapy (CCRT) is the standard treatment. However, Jemal et al. reported that one third of the patients would experience recurrence [2]. Tumor recurrence is often not detected for several months after primary therapy. Prediction of the treatment response and the long-term outcome presents a challenge in developing precise personalized care. High-risk recurrence patients can receive treatments such as additional chemotherapy and dose escalation in time by predicting reliable biomarkers.

Medical imaging, such as magnetic resonance imaging (MRI) and computed tomography, is essential in the staging of patients and guiding treatment decisions. For cervical cancer patients, the MRI provides high soft-tissue contrast and functional information, which plays a key role in the assessment of the reference standard for the pre-therapeutic study [3].

Radiomics analysis provides high-dimensional data such as tumor homogeneity and heterogeneity that cannot be identified by general visual evaluation using texture analysis in addition to the shape and volume [4,5]. Texture analysis can evaluate the position of the pixels and gray level intensity within an image using a variety of mathematical methods. Radiomics can classify the stage or histology of the tumor through the prediction of responses to chemotherapy [6] or radiotherapy [7].

Reuze et al. reported that the positron emission tomography (PET) texture analysis could predict the recurrence of cervical cancer treated by brachytherapy and chemoradiation than the maximum standardized uptake value (SUVmax) [8]. Meng et al. evaluated useful texture features extracted from T2-weighted MR images and apparent diffusion coefficient (ADC) maps for the prediction of recurrence for advanced cervical cancer patients treated with CCRT [9]. The MR-associated prediction model improved the accuracy of the prediction beyond that from the PET. In contrast, the regions of interest (ROIs) were drawn on each slice covering the whole tumor. Xie et al. showed the usability of a sub-region-based radiomics analysis in which the ROI was divided into sub-regions based on the local entropy and cluster of CT values [10]. It suggests that the number of radiomics features could be increased by adding the ROI, thus improving the prediction accuracy.

This study entailed the development of a prediction model of the recurrence for cervical cancer patients using radiomics features extracted from the extended and shrink-uterus regions on pretreatment T1- and T2-weighted MR images.

## 2. Materials and Methods

### 2.1. Patients

Eighty-nine cervical squamous cell carcinoma patients who were treated with external beam radiotherapy (EBRT) followed by intracavitary brachytherapy (ICBT) from 2003 to 2015 at our institution were reviewed. All patients provided written informed consent for treatment. The patients’ and tumor characteristics are presented in Table 1. The recurrence rate was 25% for early T (T1/T2) patients, 43% for advanced T (T3/T4) patients, and 34% for all patients. Among the patients with distant metastasis, patients with para-aortic lymph node (PAN) metastasis on imaging were included. Hiroshima University Certified Review Board approved this retrospective study (E-1656). The need for informed consent was waived owing to the retrospective nature of the study. The methodology in the current study was subjected to relevant regulations and guidelines.

### 2.2. Image Acquisition

MR images were scanned with three 1.5 T MR imaging units (Integenia Ambition, Philips; Siemens Healthcare Magnetom Avanto; Signa Excite, GE Healthcare), with a pelvic array coil for the pelvic scans. All patients were scanned using the same MR sequence, including axial T1-weighted fast spin-echo (FSE), and axial T2-weighted FSE. Patients scanned only with T1- or T2-weighted FSE with fat saturation were eliminated from the analysis. Images from 89 patients were retrospectively analyzed in an institutional review-board-approved study.

### 2.3. Treatment

#### 2.3.1. Radiotherapy

One patient was treated with ICBT alone, and 88 patients were treated with a combination of EBRT and ICBT. Three-dimensional radiotherapy planning using an X-ray beam (6–18 MV) was performed for all the patients who received EBRT. Patients without PAN metastasis received whole pelvis irradiation (WPI), and patients with PAN metastasis received extended-field irradiation. Center shielding (CS) was used in 67 patients, and boost irradiation for lymph node or parametrium regions was performed in 36 patients. The indication and dose of CS and boost irradiation were determined by the radiation oncologist based on the initial tumor size and therapeutic effect of WPI. The median EBRT dose was 50 Gy/25 fractions (range 28–66 Gy). Image-guided brachytherapy (IGBT) was performed on 20 patients. The prescribed dose of ICBT was 6 Gy to point A (a point 2 cm cranial from the external cervical os and 2 cm lateral from the tandem) in two-dimensional treatment planning and to D90 (minimum dose to 90%) of high-risk clinical target volume (the residual tumor at the time of ICBT and the whole uterine cervix) in IGBT. The fractions of ICBT depended on the CS dose, and the median ICBT dose was 18 Gy/3 fractions (range 6–30 Gy/1–5 fractions). The median overall treatment time was 44 days (range 30–58 days). The most common treatment schedule was as follows: WPI 40 Gy/20 fractions, CS 10 Gy/5 fractions, and ICBT 18 Gy/3 fractions.

#### 2.3.2. Chemotherapy

Sixty-eight patients received concurrent chemotherapy. The selection of the chemotherapeutic regimen and reduction of chemotherapeutic dosages were determined according to the hospital’s protocol and the physician’s judgment. The regimens of the chemotherapeutic were as follows: weekly cisplatin in 33 patients, weekly nedaplatin in 21 patients, intraarterial chemotherapy in 10 patients, and paclitaxel/cisplatin in two patients, irinotecan/cisplatin in one patient, and paclitaxel in one patient.

### 2.4. Radiomics Analysis

**Figure 1:**
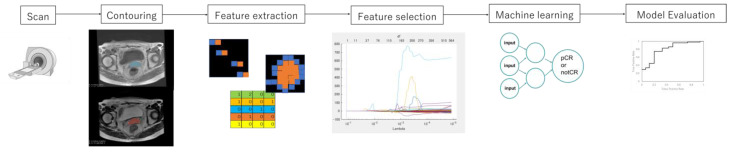
The process of the prediction model with radiomics analysis and machine learning.

**Figure 2:**
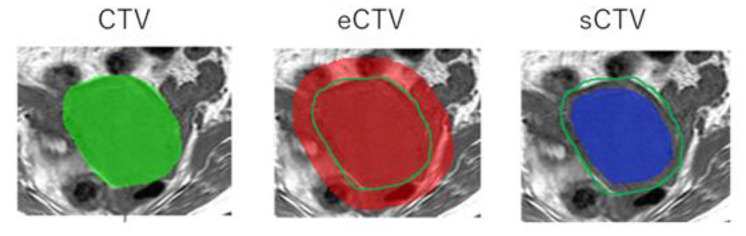
An example of the segmentation of CTV, extended-CTV (eCTV), and shrink-CTV (sCTV).

The acquisition process of the MR images to the prediction model is shown in Figure 1. The proposed radiomics model was designed as a Transparent Reporting of a Prediction Model for Individual Prognosis or Diagnosis type 2a [11]. The T1- and T2-weighted MR images were transferred to a medical image computing tool (3D Slicer, www.slicer.org, accessed on 1 January 2020) [12]. Figure 2 shows an example of the segmentation. A uterus that included the primary tumor was defined as clinical target volume (CTV) and was manually segmented on the axial T1- and T2-weighted MR images. The segmentations were performed by one or two radiation oncologists, including one expert radiation oncologist. Then, the extended-CTVs were generated by adding 5-, 10-, and 20 mm margins from the CTV, which were defined as eCTV5, eCTV10, and eCTV20, respectively. Moreover, shrink-CTVs were generated by adding 5 mm and 10 mm margins from the CTV, which were defined as sCTV5 and sCTV10. The radiomics features were extracted with an open-source package in Python, Pyradiomics software [13]. A detailed list of the radiomics features is shown in Table 2 and Table 3.

We extracted 13 shape radiomics features, 21 first-order radiomics features, 50 quantitative radiomics features, and 93 texture radiomics features. Additionally, the radiomics features were extracted from the wavelet filters with high-pass and low-pass filters. The wavelet filter was decomposed in the x, y, and z directions. A total of 837 radiomics features were extracted for each segmentation.

### 2.5. Prediction Model

In this study, all loco-regional recurrence and distant metastasis after RT were regarded as recurrence and were used to examine the prediction model. The clinical patient data were updated in May 2020, and the median follow-up time was 59 months (range, 1–160 months). Recurrence was observed in 30 of 89 patients at the last follow-up.

The least absolute shrinkage and selection operator (LASSO) regression model was used with MATLAB code to prevent overfitting [14,15]. The most significant predictive features were selected with the LASSO regression, which reduces the dimension from among all the candidate features in the training dataset.

**Figure 3:**
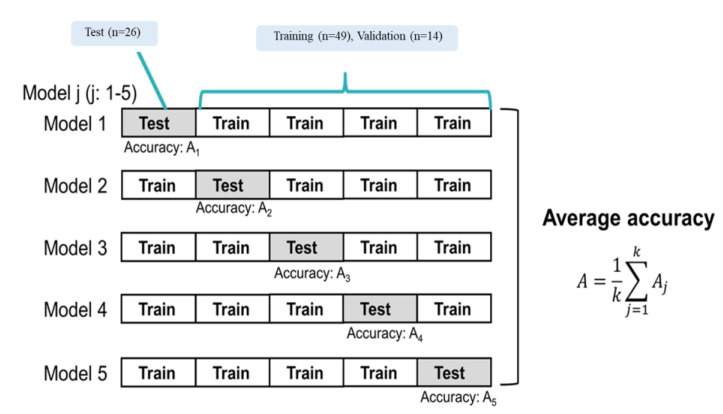
Training and testing with five-fold cross-validation.

We classified recurrence patients and non-recurrence patients with machine learning (ML) classifiers. The recurrence and non-recurrence patients were labeled as 1 and 0, respectively. The ML classifiers used a neural network (NN) with rectified linear unit activation and 10 hidden layers. All patients were randomly divided into a training set (49 patients), a validation set (14 patients), and a testing set (26 patients). The prediction model was constructed with the five-fold cross-validation method, as shown in Figure 3. The predictive performance was evaluated using the area under the curve (AUC) from the receiver operator characteristic (ROC) curve, accuracy, sensitivity, and specificity.

## 3. Results

**Figure 4:**
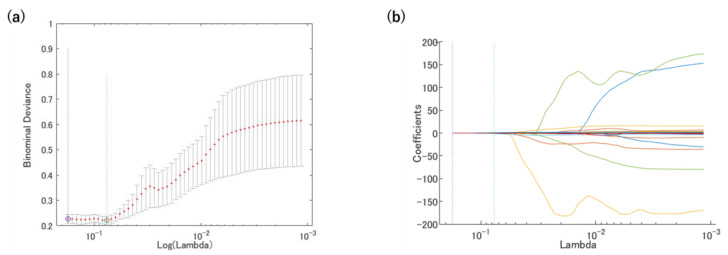
Radiomics features were selected using the LASSO regression. (**a**) Tuning penalization parameter (λ) and minimum criterion in the LASSO model. The binomial deviance was plotted against log(λ). (**b**) LASSO coefficient profiles of the 4185 radiomics features. The green line showed the optimal lambda in the LASSO analysis with the least partial likelihood of deviance.

A total of 5022 features were extracted from the T1- and T2-weighted MR images. The T1- and T2-weighted MR images were ultimately reduced to 25 and four features, respectively, with the LASSO regression model, as shown in Figure 4 and Table 4. The following features were extracted from the T1-weighted MR image: one feature from the CTV, two features from the eCTV5, seven features from the eCTV20, five features from the sCTV5, and ten features from the sCTV10. The following features were extracted from the T2-weighted MR image: one feature from the CTV, one feature from the eCTV20, one feature from the sCTV20, and one feature from the sCTV10. Most of the features from the wavelet filter were extracted. The T1-weighted MR image had more features used for the prediction model than the T2-weighted MR image. The features of the low pixel number at the center region of the uterus for the T1-weighted MR image indicate low blood flow. The radiomics feature of a lower pixel number and a high conformality was selected from the T1- and T2-weighted MR images from the shrink-CTV (sCTV) analysis. The area of the low pixel number and the conformality was larger with the T1-weighted MR image for the recurrence group compared to the T2-weighted MR image. Moreover, the mean value of the low pixel number was small with the T2-weighted MR image for the recurrence group. From the extended-CTV, the features that showed a nonuniformity, asymmetric distribution of the pixel values, and a larger volume with a high pixel number were selected.

**Figure 5:**
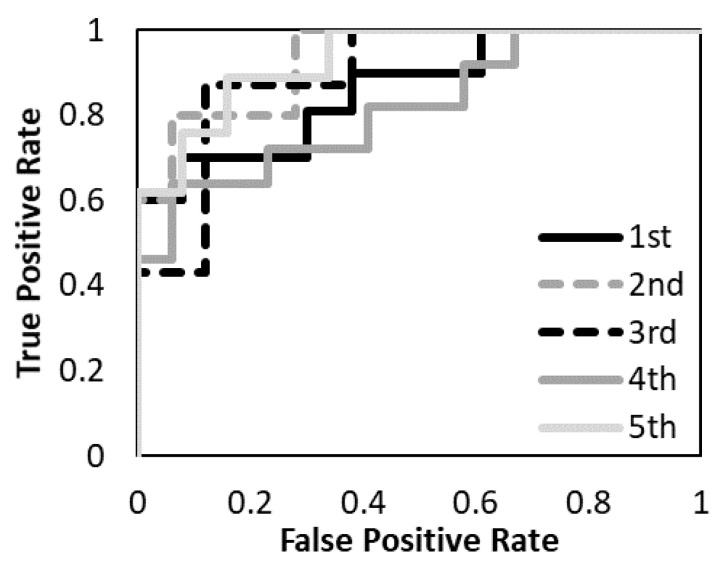
The performance of the predictive model with the T1-weighted MR image was evaluated according to the ROC metrics.

**Figure 6:**
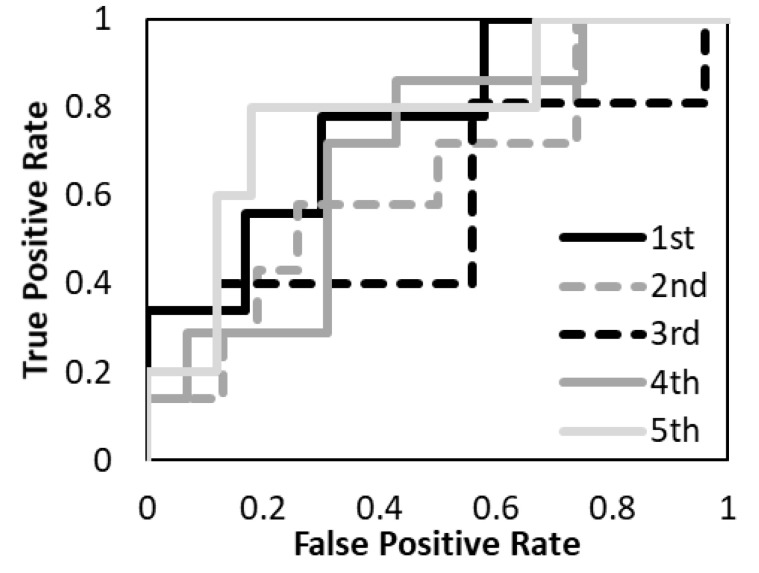
The performance of the predictive model with the T2-weighted MR image was evaluated according to the ROC metrics.

**Figure 7:**
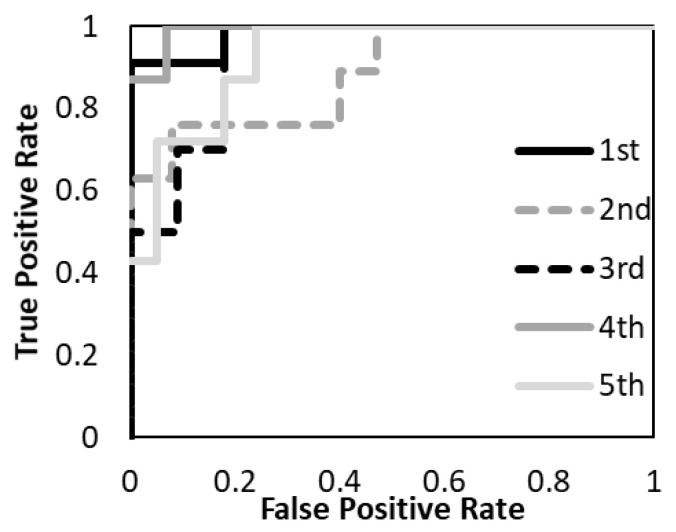
The performance of the predictive model with T1- and T2- weighted MR images was evaluated according to the ROC metrics.

The prediction models with the T1-weighted MR images, the T2-weighted MR images, and the combination of the T1-and T2-weighted MR images were evaluated. Figure 5, Figure 6 and Figure 7 show the validation of the performance of the predictive models according to ROC metrics with five-fold cross-validation. Table 5 shows the results of the accuracy, sensitivity, specificity, and AUC for the training and testing data.

The average accuracy of the five models for the testing data was 81.8% with T1-weighted MR image, 72.2% with T2-weighted MR image, and 88.7% with a combination of T1- and T2-weighted MR images. The average AUC of the five models for the testing data was 0.89 with T1-weighted MR image, 0.69 with T2-weighted MR image, and 0.94 with a combination of T1- and T2-weighted MR images. The prediction model with a combination of T1- and T2-weighted MR images had higher accuracy and AUC. The prediction model with a T1-weighted MR image had higher accuracy and AUC than the T2-weighted MR image. The specificity of the prediction model with T2-weighted MR images was under 40% for both training and testing data.

## 4. Discussion

The radiomics approach uses image-based features as the imaging biomarker for the prediction of the grade of the tumor, treatment response, and side effects of treatment. Past studies (Ho et al. and Reuze et al.) have reported that the PET texture analysis could predict the recurrence of cervix cancer based on SUVmax [8,16]. The AUC of the prediction model with PET texture analysis was 0.75 according to Ho et al. and 0.76 according to Reuze et al.; Mengal et al. improved the accuracy of the prediction of recurrence of advanced cervical cancer patients treated with concurrent chemoradiotherapy using the texture features extracted from T2-weighted MR image and apparent diffusion coefficient (ADC) maps [9]. The AUC of the prediction model with the support vector machine was 0.89.

Our study demonstrates the potential of radiomics analysis using T1- and T2-weighted MR images to predict the recurrence of cervix cancer after radiotherapy. The current study improved the accuracy of the prediction model of T1- and T2-weighted MR images with the NN. The PET is more infrequently used clinically than the MR image and the MR image has relatively more predictors.

Sun et al. showed a potential for the prediction of the clinical response to neoadjuvant chemotherapy using the radiomics analysis of combining the intratumoral and peritumoral regions on the pretreatment T1- and T2-weighted MR images [17]. The current study investigated the usability of the radiomics model based on pretreatment T1- and T2-weighted MR images for the prediction of recurrence after radiotherapy. In addition to the CTV, the features of the segmentation of the shrink-CTV and extended-CTV were selected for the prediction model. The extended CTV could extract the features in and the boundaries of the tumor, which allows us to detect its associations with metastases within the microenvironment [18].

Mayr et al. investigated the correlation of the dynamic T1-weighted MR image for the prediction of tumor control in patients treated with radiotherapy for advanced cervical cancer by pixel-by-pixel statistical analysis [19]. The dynamic MRI contrast enhancement can assess the regional variation in tumor microcirculation and facilitate a better assessment of low perfusion regions within tumors. They revealed poor blood supply and hypoxia as contributing factors to radiation therapy failure. Other studies also support the relationship between tumor hypoxia and dynamic MRI contrast enhancement [20,21,22]. Kjersti et al. divided the high and low signals on the dynamic MRI contrast images and analyzed the relation between signals and the prognosis [22]. They showed that the low signal enhancement was a biomarker of poor prognosis.

The current study performed the radiomics analysis with T1- and T2-weighted MR images, not the dynamic MRI contrast enhancement image. The shrink-CTV was mostly limited to the primary tumor region. In the shrink-CTV, the distribution of small dependence with lower pixel values was larger with the T1-weighted MR image and smaller with the T2-weighted MR image. This may suggest that the central tumor region has low blood flow. Thus, the radiomics feature can detect the hypoxia region by poor blood supply without a dynamic contrast-enhanced MR image. The blood oxygenation level-dependent (BOLD) response is sensitive to tumor vascular oxygenation [23,24,25]. Hallac et al. demonstrated that BOLD MRI examination is a potentially valuable biomarker of oxygenation [24]. Although the BOLD is sensitive to vascular oxygenation, tissue oxygen level-dependent contrast with T1-weighted MR image has the potential to more directly reflect tissue oxygenation [25,26]. Matsumoto showed the possibility to monitor changes in T1 that are related to changes in pO2 using a T1-weighted spoiled gradient echo image [25]. Moreover, O’Connor et al. showed a significant response in T1 of cervical cancer in response to oxygen breathing [25]. Zhou et al. examined T1 and T2 responses to oxygen gas breathing challenge albeit with respect to prostate cancer [26]. They identified two principal components representing 49% and 29% respectively. The current study showed a high prediction ability using the radiomics features. Thus, the radiomics analysis has a great potential to extract the biomarkers for the outcome of cancer treatment such as oxygenation from pretreatment T1- and T2-weighted MR images. In the present study cohort, the recurrence rates were lower for early T (T1/T2) patients than advanced T (T3/T4) patients. Lee, et al. reported a significant correlation between VEGF expression and tumor size, and deep cervical invasion [27]. Advanced cervix cancer causes more angiogenesis. Angiogenesis increases the vessel to provide oxygen and nutrients to the growing tumor tissue that expands rapidly; however, more cancer cells means more demand causing even more hypoxia. The tumor ends up being highly hypoxic with dysfunctional vasculature [28]. Although the validation of the correlation between these features and blood flow and hypoxia is required in the future, we believe that the results of this study focused on T1-weighted MR images are of great significance. In addition to hypoxia in the tumor, the poor prognosis for cervical cancer has been associated with high interstitial fluid pressure (IFP). Simonsen et al. showed that the prognosis is less affected by tumor hypoxia and strongly affected by the IFP [9]. We will reveal the tumor microenvironment from the radiomics feature in further study.

There were several limitations in the current study. The study was conducted at a single institution with a limited number of cervix cancer patients. The study did not set the exclusion criteria according to the prescription dose and ICBT alone. We consider it necessary to examine the universal prediction model with a large number of cases in a multicenter and perform a sub-group analysis. The current study used multiple MRI devices. A further study will be performed to reveal the robustness of the radiomics features between these devices. The prediction model was proposed only with pretreatment MR images. Meng et al. improved the prediction of the recurrence of cervix cancer using MR images during treatment [29]. The changes in radiomics features from pretreatment and during treatment, called delta-radiomics features, have been investigated for their prognostic potential in cancer [30,31,32]. In the future, we will reveal the correlation between the radiomics features and the biological effect and construct a highly versatile predictive model.

## 5. Conclusions

The radiomics analysis with T1- and T2-weighted MR images can accurately predict the recurrence of cervix cancer after radiotherapy. The variation of the distribution and the difference of the pixel number at the peripheral and the center were important predictors.

## Figures and Tables

**Table 1:** Patient and tumor characteristics.

Age (Years)	Median (Range)	63 (30–85)
PS	0	71
	1	14
	2	4
	3	0
Histology	Squamous	89
T factor (UICC-8th)	1a	1
	1b	8
	2a	1
	2b	37
	3a	0
	3b	36
	4a	6
N factor	0	43
	1	46
M factor	0	76
	1	13

**Table 2:** Feature type and associated features.

Feature Type	Methods	Feature Name
Morphology-based	Shape	Maximum 3D diameter
		Maximum 2D diameter slice
		Sphericity
		Minor axis
		Elongation
		Surface volume ratio
		Volume
		Major axis
		Surface area
		Flatness
		Least axis
		Maximum 2D diameter column
		Maximum 2D diameter row
First or-der-based	Histogram	Interquartile range
		Skewness
		Uniformity
		Median
		Energy
		Robust mean absolute deviation
		Mean absolute deviation
		Total energy
		Maximum
		Root mean squared
		90 percentile
		Minimum
		Entropy
		Range
		Variance
		10 percentile
		Kurtosis
		Mean
Texture-based	GLCM	Joint average
		Sum average
		Joint entropy
		Cluster shade
		Maximum probability
		Idmn
		Joint energy
		Contrast
		Difference entropy
		Inverse variance
		Difference variance
		Idn
		Idm
		Correlation
		Autocorrelation
		Sum entropy
		Sum squares
		Cluster prominence
		Imc2
		Imc1
		MCC
		Difference average
		Id
		Cluster tendency
	GLSZM	Gray level variance
		Zone variance
		Gray level non-uniformity normalized
		Size zone non-uniformity normalized
		Size zone non-uniformity
		Gray level non-uniformity
		Large area emphasis
		Small Area high gray level emphasis
		Zone percentage
		Large area low gray level emphasis
		Large area high gray level emphasis
		High gray level zone emphasis
		Small area emphasis
		Low gray level zone emphasis
		Zone entropy
		Small area low gray level emphasis
		Gray level variance
	GLRLM	Short run low gray level emphasis
		Gray level variance
		Low gray level run emphasis
		Gray level non-uniformity normalized
		Run variance
		Gray level non-uniformity
		Long run emphasis
		Short Run high gray level emphasis
		Run length non-uniformity
		Short run emphasis
		Long run high gray level emphasis
		Run percentage
		Long run low gray level emphasis
		Run entropy
		High gray level run emphasis
		Run length non-uniformity normalized
	NGTDM	Coarseness
		Complexity
		Strength
		Contrast
		Busyness
	GLDM	Gray level variance
		High gray level emphasis
		Dependence entropy
		Dependence non-uniformity
		Gray level non-uniformity
		Small dependence emphasis
		Small dependence high gray level emphasis
		Dependence non-uniformity normalized
		Large dependence emphasis
		Large dependence low gray level emphasis
		Dependence variance
		Large dependence high gray level emphasis
		Small dependence low gray level emphasis
		Low gray level emphasis

GLCM, gray-level co-occurrence matrix; GLSZM, gray-level size-zone matrix; GLRLM, gray-level run-Length matrix; NGTDM, neighboring gray-tone difference matrix; GLDM, gray-level dependence matrix.

**Table 3:** Feature associated with the imaging filters.

Feature Type	Wavelet-Based
Methods	First-order statistic and texture of wavelet decomposition. Decomposition levels: LLL, LLH, LHL, LHH, HLL, HLH, HHL, HHH.
Feature name	First-order features
	GLCM features
	GLSZM features
	GLRLM features
	NGTDM features
	GLDM features

**Table 4:** Selected the features by LASSO Cox regression.

ROI	Filter	Feature List	
T1-weighted MR image			
CTV	wavelet-LLH	Firstorder	Skewness
eCTV5	wavelet-HLL	GLDM	LargeDependenceHighGrayLevelEmphasis
eCTV5	wavelet-HLH	GLCM	Correlation
eCTV20	Original	Shape	SurfaceVolumeRatio
eCTV20	Original	GLCM	MCC
eCTV20	wavelet-LLH	GLSZM	GrayLevelNonUniformity
eCTV20	wavelet-HLH	Firstorder	Kurtosis
eCTV20	wavelet-HHH	Firstorder	Skewness
eCTV20	wavelet-HHL	Gldm	DependenceNonUniformity
eCTV20	wavelet-HHL	Glcm	Imc1
sCTV5	wavelet-HLL	Glcm	InverseVariance
sCTV5	wavelet-HLL	Firstorder	Skewness
sCTV5	wavelet-LHL	Glszm	LargeAreaLowGrayLevelEmphasis
sCTV5	wavelet-LLH	Firstorder	Skewness
sCTV5	wavelet-HLH	Firstorder	Median
sCTV10	Original	Glszm	LargeAreaHighGrayLevelEmphasis
sCTV10	wavelet-HLL	Glcm	InverseVariance
sCTV10	wavelet-LHH	Firstorder	Mean
sCTV10	wavelet-LLH	Gldm	SmallDependenceLowGrayLevelEmphasis
sCTV10	wavelet-HLH	Firstorder	Mean
sCTV10	wavelet-HHH	Gldm	SmallDependenceLowGrayLevelEmphasis
sCTV10	wavelet-HHH	Firstorder	Skewness
sCTV10	wavelet-HHL	Firstorder	Skewness
sCTV10	wavelet-HHL	Firstorder	Mean
sCTV10	wavelet-LLL	Gldm	SmallDependenceLowGrayLevelEmphasis
T2-weighted MR image			
CTV	wavelet-HHH	Firstorder	Median
eCTV20	wavelet-HLL	Firstorder	Skewness
sCTV5	wavelet-HHH	Firstorder	Median
sCTV10	Original	Gldm	SmallDependenceLowGrayLevelEmphasis

**Table 5:** Assessment of the predictive performance of the predictive model for training and testing data with T1-weighted MR image (T1), T2-weighted MR image (T2), and the combination of T1- and T2-weighted MR images (T1&T2).

	T1		T2		T1&T2	
	Training	Test	Training	Test	Training	Test
Sensitivity	89.9	86.4	87.8	87.4	97.6	93.1
Specificity	81.7	74.9	31.3	38.1	92.2	81.6
Accuracy	87.2	81.8	67.9	72.2	95.9	88.7
AUC		0.89		0.69		0.94

## Data Availability

The data presented in this study are available on request from the corresponding author.
